# Is in vivo and ex vivo irradiation equally reliable for individual Radiosensitivity testing by three colour fluorescence in situ hybridization?

**DOI:** 10.1186/s13014-019-1444-4

**Published:** 2019-12-31

**Authors:** Theresa Mayo, Marlen Haderlein, Barbara Schuster, Anna Wiesmüller, Christian Hummel, Maximilian Bachl, Manfred Schmidt, Rainer Fietkau, Luitpold Distel

**Affiliations:** Department of Radiation Oncology, Friedrich-Alexander-University of Erlangen-Nuernberg, Erlangen, Germany

**Keywords:** Three color fluorescence in situ hybridization, Lung cancer, Rectal cancer, Individual radiosensitivity, Chromosomal aberrations, Breaks per metaphase

## Abstract

**Background:**

Individual radiosensitivity is influencing the outcome of radiation therapy. A general ex vivo testing is very work-intensive. It is of interest to see if a significant prediction concerning the sensitivity can be made by in vivo irradiation during radiation treatment.

**Methods:**

Blood samples of 274 patients with rectal cancer and 43 lung cancer patients receiving radiotherapy were examined after 2 Gy ex vivo and in vivo ionizing radiation. Chromosomes # 1, 2 and 4 were stained by the 3-color-fluorescence in situ hybridization. Chromosomal aberrations were analyzed as breaks per metaphase (B/M). The deposited energy per session was calculated for each patient.

**Results:**

Weak correlation could be found between the chromosomal aberrations ex and in vivo. Though receiving significantly smaller deposited energy during radiation therapy (RT) the lung cancer cohort displayed B/M values similar to the rectal cancer cohort. Considering the individual deposit energy differences improved slightly the correlation.

**Conclusions:**

As various factors influence the induction of chromosomal aberrations it seems not feasible to estimate individual radiosensitivity via in vivo irradiation. An ex vivo estimation of individual radiosensitivity should be preferred.

## Introduction

Individualizing cancer treatment is gaining more and more attention. In radiation therapy especially detecting radiosensitive patients is important. These have an increased risk to suffer from severe side effects like fibrosis or even limited function of organs [1, 2]. To minimize these, generally testing each individual’s radiosensitivity prior to treatment could be a solution. Not only it would decrease the rate of side effects but also the dose could be adapted accordingly. Currently the dose is chosen rather low, so the more sensitive patients do not suffer from an increased risk of severe side effects. Simultaneously the dose might not be high enough to inactivate all malign cells effectively. Not only sensitive individuals exist but also ones with increased resistance to ionizing radiation (IR), which would benefit from a dose escalation [3, 4]. Even tumor types differ in individual radiosensitivity [5]. By adjusting the dose according to individual sensitivities the prospects of healing might be increased.

Today testing is only done for individuals who already reacted particularly sensitive, or who have known predispositions for increased sensitivity, which has worked very well. Even though it would be best to identify sensitive patients prior to starting treatment this is not realizable. The individual sensitivity is often tested by scoring chromosomal aberrations in irradiated blood lymphocytes. Chromosomal aberrations are non-repaired or misrepaired DNA lesions indicating the ability of the cells to process the induced DNA damage. Most studies were able to predict increased radiosensitivity using the chromosomal aberration assays [1, 6–13]. However, these assays are too expensive and time consuming plus need an expert to score the metaphases. Other authors have shown interest in detecting correlations between chromosomal aberrations during radiation and acute clinical side effects as well [1]. Consequently, the question arises whether there is a possibility to test the patients during radiotherapy, if any sign of increased or premature sensitivity should occur.

We studied whether individual radiosensitivity can be evaluated in patients after one or three weeks of radiotherapy by simply drawing blood. Therefore, we performed a chromosomal aberration study by 3-color-fluorescence in situ hybridization (FiSH) assay on blood lymphocytes of lung cancer and rectal cancer patients. Lymphocytes were irradiated ex vivo in the G0 phase and afterwards stimulated by phytohemagglutinin. Lymphocytes progress the whole cell cycle and finally are arrested in the metaphase. Though by this G0 assay not only DNA repair of the cells is checked, but additionally correct signal transduction and cell cycle control. We compared the individual radiosensitivity after ex vivo irradiation of the blood to in vivo irradiation after one and three weeks of radiotherapeutic treatment. During In vivo irradiation deposited doses were calculated to correct the chromosomal aberrations.

## Methods

### Patient selection & chromosome preparation

Lymphocytes of 43 consecutive lung and 274 consecutive rectal cancer patients were examined *(*Table 1*).* All patients were treated by radiochemotherapy*.* Heparinized blood was drawn prior to the beginning of the radiotherapeutic treatment and on Mondays after the first week of treatment. In the lung cancer patient cohort an additional blood sample was drawn on Monday after three weeks of radiotherapy. The first sample of each individual was divided and one-half was irradiated with 2 Gy by a 6-MV linear accelerator (Oncor, Siemens, Germany). The other half was not irradiated and used to estimate the background of chromosomal aberrations. For the dose effect curve of chromosomal breakage rates blood samples were irradiated by 0.4, 0.7, 1.4 and 2.0 Gy IR using the linear accelerator. Lymphocytes were irradiated in G0 phase and afterwards stimulated by phytohemagglutinin (PHA-L pure, Cat.No. M 5030) (Biochrom, Berlin, Germany) and incubated for 48 h. 48 h incubation time was used to have exclusively lymphocytes in the first mitosis following PHA stimulation. Colcemid was used to arrest lymphocytes in the metaphase. The chromosome preparation was performed according to a standard protocol. Fluorescence in situ Hybridization (FiSH) was carried out as previously described [14–17]. Chromosomes #1, 2 and 4 were painted in red, green and yellow by fluorescent dyes and DNA was counterstained with DAPI.

**Table 1:** Patient characteristics

Lung cancer	Rectal cancer	
n	43	274
Gender (%) *		
Male	34 (79)	76 (28)
Female	9 (21)	198 (72)
Age (years)		
Mean age	65.2	63.4
Range	49–83	23–87
Chemotherapy (agent)		
5-FU		52
5-FU/Platin-derivative	4	189
Cisplatin in combination with other agents	17	
Carboplatin (solo or in combination)	14	
Other Chemotherapy	5	20
No Chemotherapy	3	13
Staging		
I	1	3
II	4	30
III	18	176
IV	11	37
unknown	9	28

### Image acquisition and analysis

Chromosomal aberrations were scored using a fluorescence microscope (Zeiss, Axioplan 2, Göttingen, Germany) and Metasystem software (Metafer 4 V3.10.1, Altlussheim, Germany). Metaphases were searched automatically and an image of each metaphase was acquired. Images were analyzed with the help of an image analyzing software (Biomas, Erlangen, Germany) and all chromosomal aberrations were scored by the number of underlying DNA breakages according to Savage and Simpson [16]. Chromosomal aberrations were scored as breaks per metaphase (B/M). The average number of metaphases analyzed were 351/305 rectal/lung for the unirradiated samples and 171/157 rectal/lung metaphases for the irradiated samples. Background rates of none irradiated samples were subtracted from the rates of the 2 Gy irradiated samples. Samples analyzed after one and three weeks were not corrected for background rates.

### Estimated average dose

Dose values and volumes to calculate the estimated average dose by a treatment were extracted from the treatment planning software (TPS) Pinnacle (Philips Radiation Oncology Systems, Fitchburg, WI, USA) (Fig. 2C).

### Statistical analysis/methods

The statistical analysis of the data was performed with SPSS Statistics 21 (IBM, Armonk, NY, USA). Statistical significance was tested by the T- and the Levene-test. The Graphs were plotted with Excel (Microsoft Corporation, Redmond, WA, USA) and SPSS Statistics 21.

## Results

**Fig. 1:**
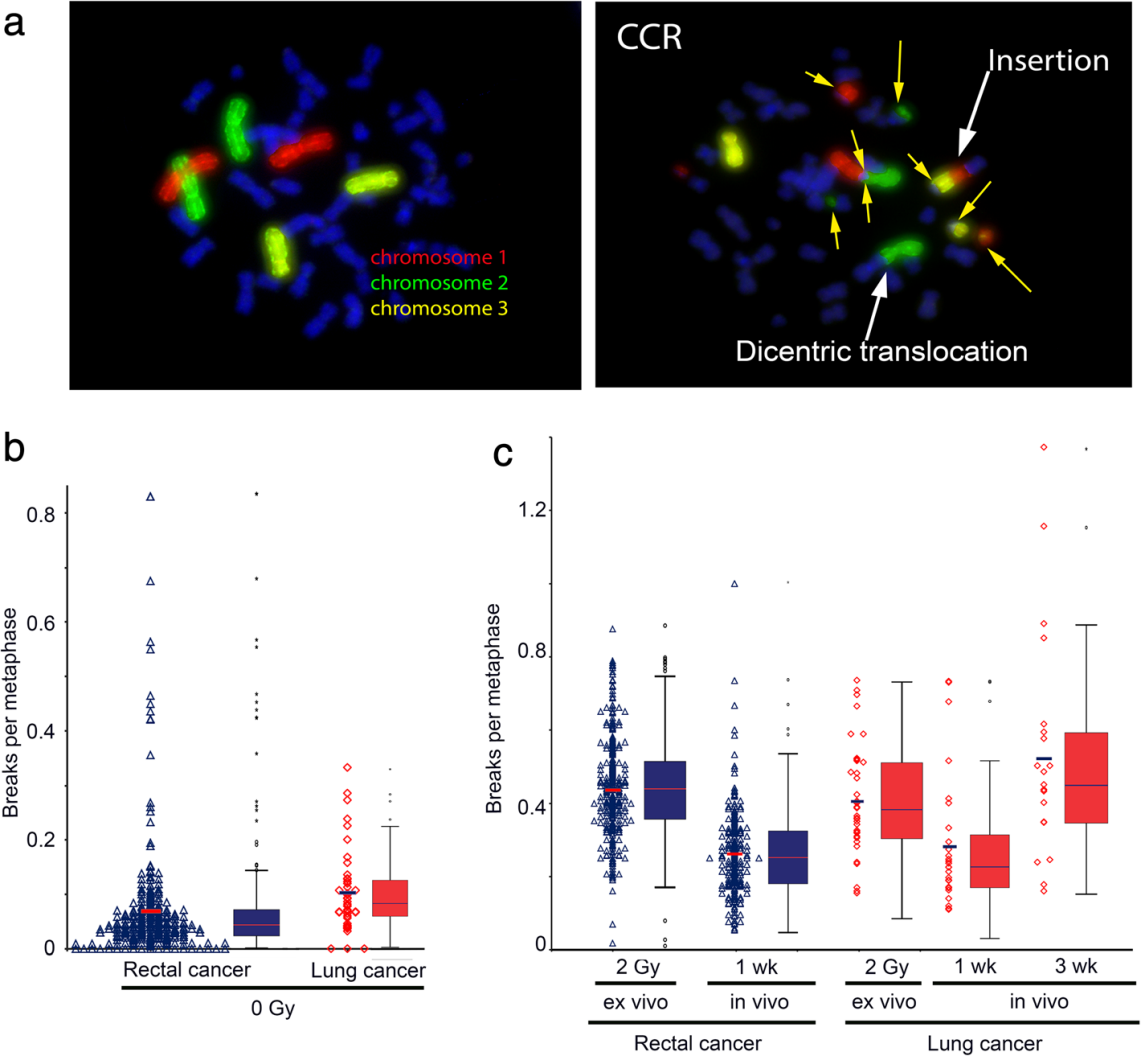
3-color-Fluorescence in situ hybridization. Metaphase spreads of human blood lymphocytes with chromosomes # 1 (red), # 2 (green) and # 4 (yellow) stained with a chromosome specific probe. DNA was counterstained with DAPI (blue). (**a**) Normal metaphase spread in comparison to a metaphase spread with complex aberrations in almost every chromosome (CCR), in sum scored with 10 breaks per metaphase. (**b**) The yellow arrows indicate chromosomal breaks and aberrations. Individual background B/M rates for both cancer cohorts (**c**) as well as the breakage rates after ex vivo and in vivo IR

We studied individual radiosensitivity using a G0 three Color in situ hybridization assay (Fig. 1a). Chromosomal aberrations in the three largest chromosomes were rated as breaks per metaphase (B/M) in dependence of the aberration types. We studied a rectal cancer cohort including 274 patients as well as a lung cancer cohort including 43 patients (Table 1). The background breakages are 0.069 ± 0.107 B/M for rectal cancer patients and 0.103 ± 0.0753 B/M for lung cancer patients. Breaks are slightly higher in the lung cancer cohort (*p* = 0.049). There are a few individual outliers with partly extremely high values (Fig. 1b).

The values of the blood samples irradiated with 2 Gy ex or in vivo do not significantly differ between the rectal and lung cancer patients (*p* > 0.16). The in vivo irradiated samples are derived from cancer patients who were irradiated five consecutive times with a single dose of 1.8 Gy in one week and blood was drawn on Monday prior to the next fraction. These breakage rates were clearly lower compared to the samples irradiated ex vivo with 2 Gy (*p* = 0.001 rectal cancer, *p* < 0.001 lung cancer) (Fig. 1c). Samples after three weeks of irradiation were only evaluated in the lung cancer patients’ cohort. The breakage rates nearly doubled when compared to the 1 week in vivo rates (*p* < 0.001) and had a tendency to exceed the ex vivo irradiation rates (*p* = 0.130).

**Fig. 2:**
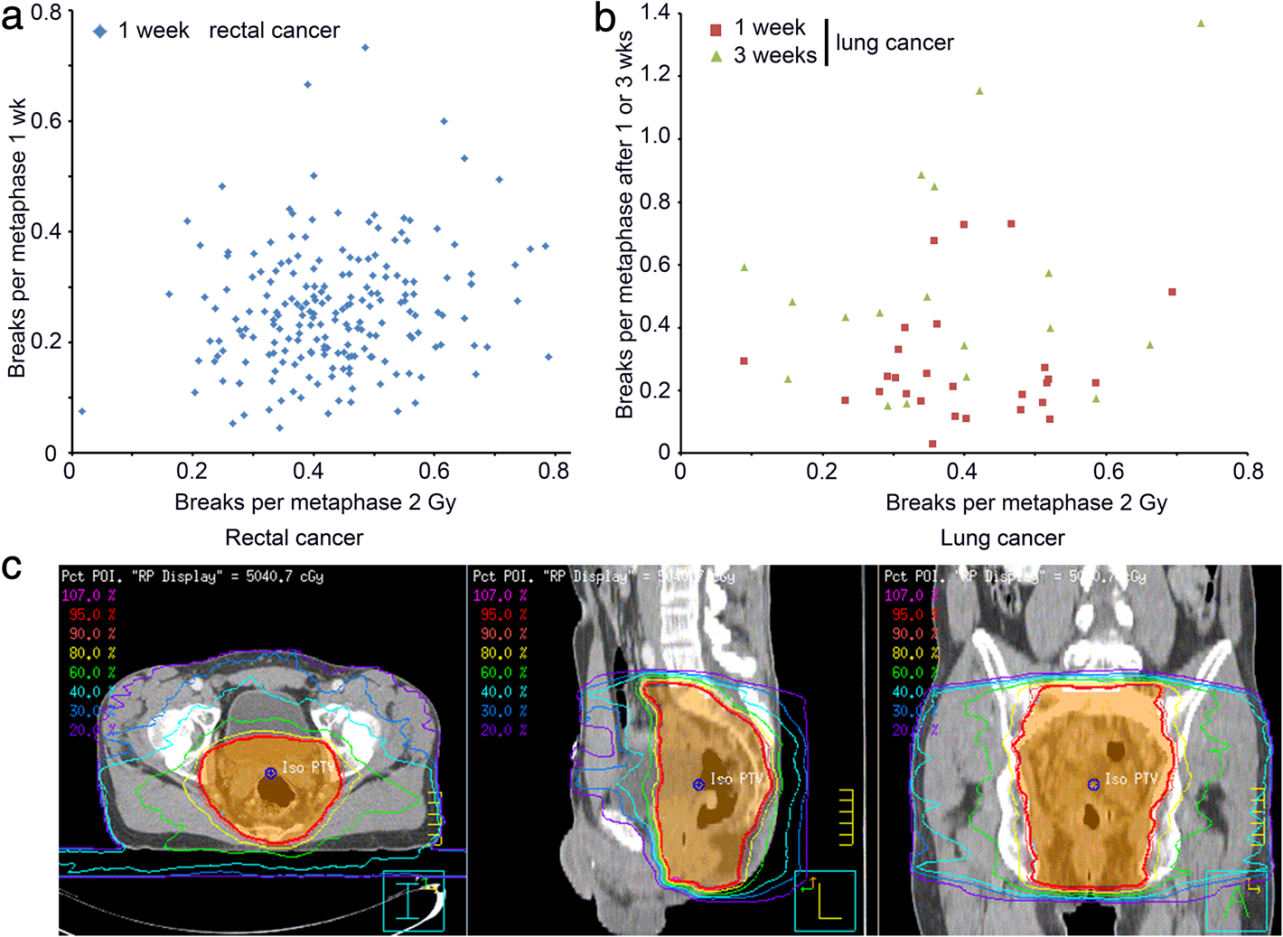
(**a**) Individual radiosensitivity expressed as breaks per metaphase after 2 Gy ex vivo irradiation correlated with breaks per metaphase after one or three weeks in vivo irradiation in the rectal cancer and (**b**) the lung cancer cohort. (**c**) Treatment planning images from one rectal cancer patient with the particular isodose lines marked in different colors

The differences in individual radiosensitivity should influence the B/M values in general and especially during therapy. Thus we were interested whether the in vivo data after one and three weeks correlated with the ex vivo data, which we currently use as a marker for individual radiosensitivity. In the rectal cancer cohort (1wk: r = 0.118, *p* = 0.079) as well as in the lung cancer cohort (1wk: r = 0.081, *p* = 0.687; 3wks: r = 0.285, *p* = 0.252) there was no significant correlation between in vivo and ex vivo radiosensitivity testing (Fig. 2a, b).

**Fig. 3:**
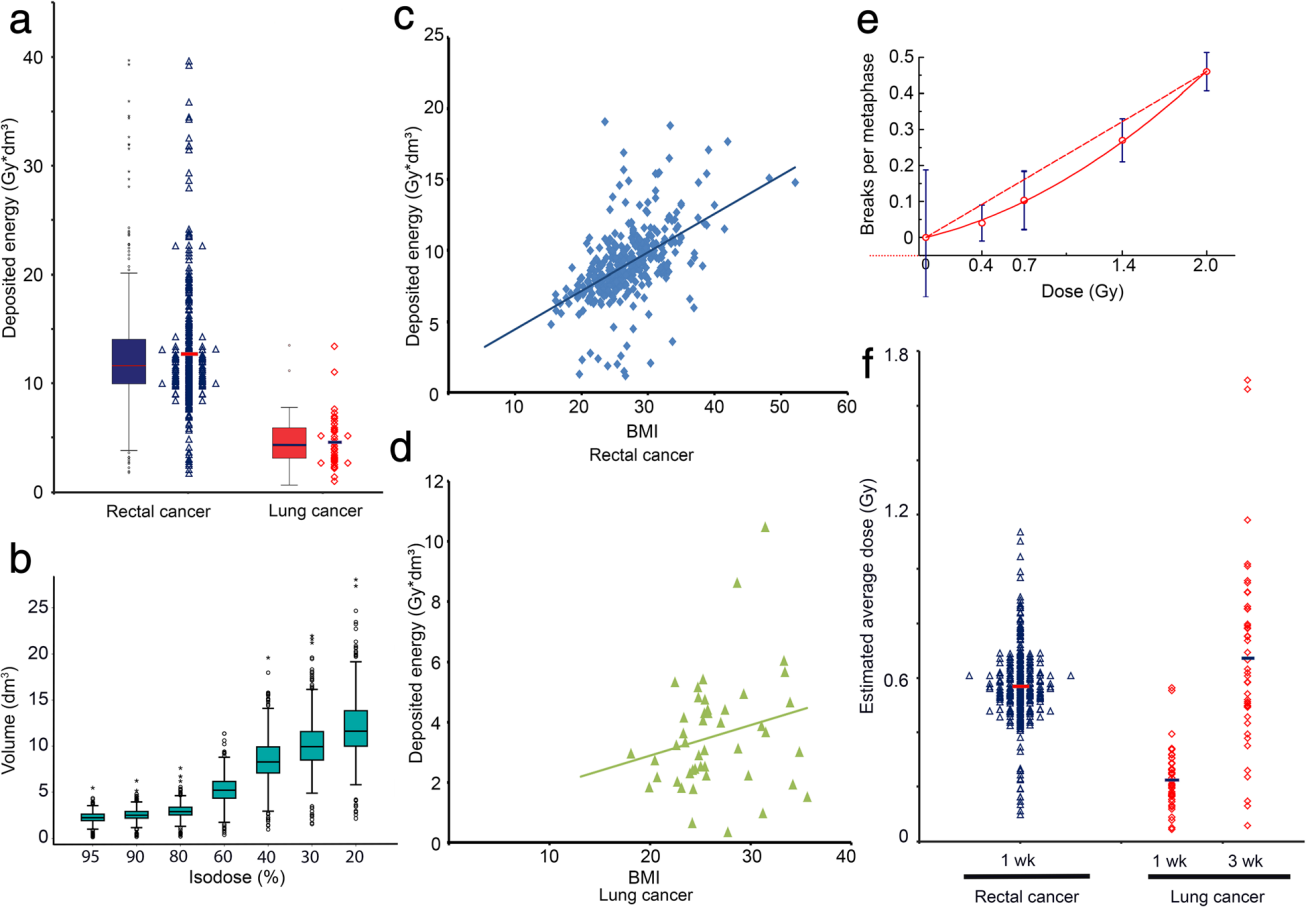
(**a**) The deposited energy distribution is calculated for both cohorts. (**b**) The range of isodose volumes within the particular isodoses in the rectal cancer cohort. (**c**) Correlation of the individual BMI value with the deposited energy for rectal and (**d**) lung cancer patients. (**e**) Chromosomal breakage rates after ex vivo IR using different IR doses. Linear dose-response curve (solid line) and the calculated linear-quadratic adjustment line (dashed line). The linear quadratic function was used to correct the formed chromosomal aberrations in dependence of the respectively dose volume. Distribution of the estimated average dose. The estimated average dose is the deposited energy of the different isodose volumes and is corrected for the chromosomal aberrations efficiency. (**f**) Additionally it is multiplied by the number of fractions and divided by the body weight of each patient

The in vivo irradiated data must be clearly dependent on the deposited energy every patient received by the radiation and on the size (volume) of the patient. To consider this we were interested to see the variation of the deposited energy in these patients. Using the irradiation planning software (Fig. 3a) we extracted the isodose volumes and calculated an estimation of the total deposited energy for each patient:

$$ {E}_{dep}=\sum \limits_i{D}_i\bullet {m}_i, $$

where the D_i_ are the isodose levels and the m_i_ the isodose volumes times the density ρ of the tissue, both extracted from the TPS pinnacle. Terms are additionally explained in Table 2.

$$ {E}_{dep}=\left[0.95\bullet 1.8\  Gy\bullet {V}_{95\%}+0.9\bullet 1.8\  Gy\bullet \left({V}_{90\%}-{V}_{95\%}\right)+0.8\bullet 1.8\  Gy\bullet \left({V}_{80\%}-{V}_{90\%}\right)+\dots \right]\bullet \rho, $$

where V_x_ is the volume inside the x% isodose line. As an approximation we set the density to 1 kg/dm^3^.

The deposited energies are spread widely in both cohorts (Fig. 3a). Within the rectal cancer patients the deposited energy is clearly higher when compared to the lung cancer patients (*p* < 0.001, 9.1 vs. 3.5 Gy*dm^3^). The volumes are smallest for the 95% isodose volume and highest, as well as wider spread, for the low dose volumes (Fig. 3b). As the later may be dependent on the individual patients’ physique we investigated the correlation between BMI and the deposited energy (Fig. 3c, d). For the rectal cancer cohort there is a correlation (r = 0.523; *p* < 0.001), while for the correlation between BMI and deposited energy there is only a tendency in lung cancer patients (r = 0.228, *p* = 0.128). Mainly the low dose isodose volumes vary, whilst they only amount for a low percentage of the whole dose, especially within the rectal cancer patients (8.4%) (Table 3). In low dose regions however chromosomal aberrations are formed by lower efficiency when compared to higher doses (Fig. 3e). Considering this, every isodose volume was multiplied with a correction factor derived from the dose effect curve of chromosomal aberrations (Fig. 3e) to get the effective deposited energy:

$$ {E}_{dep}^{eff}=\left[0.95\bullet 1.8\  Gy\bullet {V}_{95\%}\bullet {cf}_{0.95\bullet 1.8}+0.9\bullet 1.8\  Gy\bullet \left({V}_{90\%}-{V}_{95\%}\right)\bullet {cf}_{0.9\bullet 1.8}+0.8\bullet 1.8\  Gy\bullet \left({V}_{80\%}-{V}_{90\%}\right)\bullet {cf}_{0.8\bullet 1.8}+\dots \right]\bullet \rho, $$

where cf_x_ is the dose dependent chromosomal aberration correction factor of dose x.

**Table 2:** Definitions, calculations and interpretations of introduced terms

deposited energy	E_dep_	Calculation: Is the volume inside the isodose levels multiplied by the density ρ and the assigned dose and summed up for the isodose levels 20, 30, 40, 60, 80, 90 and 95%. Interpretation: An estimation of total absorbed radiation energy.
chromosomal aberration correction factor	cf	Calculation: Is derived from a linear quadratic fit for the induction of chromosomal aberrations in dependence of dose. Interpretation: chromosomal aberrations are non-linear induced in dependence of dose. In low dose areas relative lower amounts of aberrations are induced compared to higher dose regions.
effective deposited energy	${E}_{dep}^{eff}$	Calculation: Is calculated according to the deposited energy while including the chromosomal aberration correction factor (cf) for each isodose level. Interpretation: The dose dependence of the induction of chromosomal aberrations is included.
estimated average dose	EAD	Calculation: Is the deposited/absorbed radiation energy of an individual divided by the mass of this individual. Interpretation: It should reflect the exposed average dose of the blood and respectively the blood lymphocytes.
normalized breaks per metaphase	B/M_norm_	Calculation: The B/M of an individual is multiplied by the average EAD of the whole cohort divided by the EAD of the individual. Interpretation: The B/M values of all individuals are normalized to the same dose.
radiosensitivity factor	--	Calculation: The breaks per metaphase of an individual divided by the average B/M of all individuals for the 2Gy ex vivo irradiation. Interpretation: A factor for each individual giving the deviation from the average radiosensitivity expressed as chromosomal aberrations.

A higher effective deposited energy yields to a higher number, a larger size/weight of the patient, however, to a lower number of chromosomal aberrations per volume. To compensate these influences of different deposited energies and the different weights of the patients the averaged dose of the blood is estimated by:

$$ EAD=\frac{E_{dep}^{eff}}{M}, $$

where EAD is the estimated averaged dose and M the mass of the patient.

This parameter theoretically equals the average dose every lymphocyte approximately receives during one fraction. As the blood samples are drawn after one or three weeks of therapy the patients receive a cumulative dose of five or fifteen times this estimated average dose. This was calculated for both cohorts (Fig. 3f). Even though the dosages were corrected it is still apparent that the rectal cancer cohort receives a clearly higher estimated average dose (*p* < 0.001) caused by the irradiation of overall larger volumes.

To normalize the B/M values of all patients to the same dose we apply the following normalization factor on the B/M value:

$$ B/{M}_{norm}=B/M\bullet \frac{\overline{EAD}}{EAD}, $$

where $\overline{EAD}$ is the mean of *EAD* of the whole cohort.

**Fig. 4:**
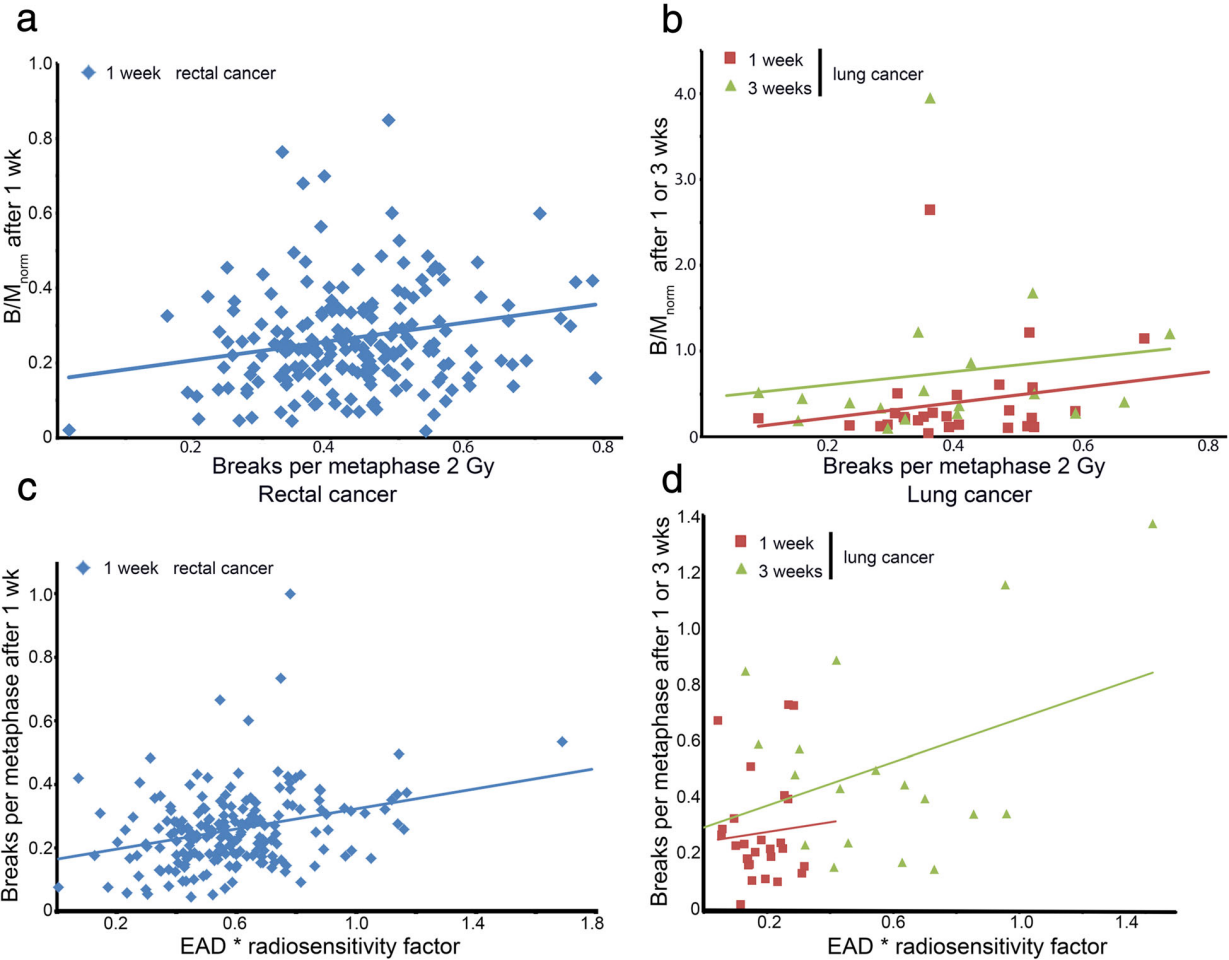
Correlation of the individual radiosensitivity after 2 Gy ex vivo IR with the in vivo IR B/M values divided by the estimated average dose (EAD) factor for (**a**) rectal and (**b**) lung cancer patients. Correlation of the estimated average dose multiplied by the radiosensitivity factor with the in vivo breaks per metaphase for the (**c**) rectal and (**d**) lung cancer cohort

If the in vivo B/M number would be a good measurement for radio sensitivity, the B/M_norm_ should lead to a strong correlation with the B/M after 2 Gy ex vivo. Within the lung cancer cohorts, the values after one and three weeks did not correlate significantly, though there is a tendency for a positive correlation especially after the first week (1 week: r = 0.209, *p* = 0.296, 3 weeks: r = 0.013, *p* = 0.961). In contrast the rectal cancer patients correlates (r = 0.194, *p* = 0.006) (Fig. 4a, b).

Analogue, we expected that the estimated average dose of each patient multiplied with a radiosensitivity factor estimated by the 2 Gy ex vivo measurements should correlate strongly with the in vivo B/M.

$$ Radiosensitivity\ factor=\frac{B/{M}_{ex\  vivo}}{\overline{B/{M}_{ex\  vivo}}}, $$

where $\overline{B/{M}_{ex\  vivo}}$ is mean of B/M_ex vivo_ of the whole cohort.

The *EAD multiplied by the radiosensitivity factor* tends to correlate with the in vivo irradiation B/M values. Within the lung cancer cohort there is no significant correlation (1 wk.: r = 0.363, *p* = 0.139; 3 wks: r = 0.073, *p* = 0.717) while the rectal cancer cohort demonstrates a weak correlation (r = 0.301, *p* < 0.001) (Fig. 4c, d).

## Discussion

We investigated estimating radiosensitivity after the beginning of radiotherapeutic treatment via in vivo irradiated lymphocytes. In this study we used the 3-color-FiSH technique to evaluate and compare chromosomal aberrations in two cancer cohorts. Numerous methods are described to test for increased sensitivity to radiation but in the majority of studies chromosomal aberrations were able to predict individual radiosensitivity [1, 6–12]. Still most of the chromosomal aberrations occurring in the painted chromosomes can be evaluated by the 3-color-FiSH [15, 18–23]. In addition this assay is very sensitive also in correlating the aberrations to acute radiation side effects [14, 24, 25]. Nevertheless, the correlation between individual radiosensitivity estimation after in vivo irradiation and the estimation after 2 Gy ex vivo irradiation was weak.

**Table 3:** Distribution of dose

Isodose (%)	Cancer cohort	0.95–0.8	0.6–0.4	0.3–0.2
Proportion of the whole deposited energy	Lung cancer	45.4%	37.3%	17.3%
	Rectal cancer	51.1%	40.5%	8.4%

The individual radiosensitivity evaluated by the 2 Gy ex vivo approach did not indicate considerable differences between the two cancer cohorts. Though the deposited energy within the lung cancer patients was only half the energy measured within the rectal cancer patients, both cohorts had the same amount of in vivo chromosomal aberrations after one week. The reason may be that the lungs hold a higher blood volume and blood flow then the pelvis, even though the lungs have a lower density [26]. Additionally, a higher percentage of the pelvis consists of fat with less blood circulation. We assume that this may be the main reason for the differences concerning deposited energy and chromosomal aberrations among our cohorts. Remarkably is that in vivo irradiation depends greatly on the blood circulation in the irradiated area and can lead to astonishing differences in DNA damage and chromosomal aberrations.

Another limitation factor is the general physique that has great influence on the deposited dose, especially within the rectal cancer cohort. The 95% isodose enclosing the tumor is similar for most patients whilst the lower isodoses are dependent on physical corpulence meaning mostly the amount of abdominal fat. The higher the BMI the greater the deposited energy whilst the lymphocytes do not necessarily receive a comparable higher dose. However, the accuracy of the isodose volumes in the planning software is lower for the low isodose volumes when compared to higher isodose volumes. Yet only 8.4% respectively 17.3% of the deposited energy is deposed in the 30% and lower isodose volumes and therefore should only play a minor role in inducing chromosomal aberrations.

A further factor influencing the formation of chromosomal aberrations is the duration of irradiation. Ex vivo irradiation lasts about 1 min, while the actual therapy session takes several minutes (approx. 10 min) and hence the dose rate is lower. The idea that during one radiotherapy session the lymphocytes cross the different isodose volumes and receive roughly the estimated dose is only a model picturing the ideal case. Usually every cell will receive a slightly different dose. Furthermore, the extracted isodose volumes do not strictly contain the exact percentage of the whole dose but display more of a continuous transition of doses. This makes a dose prediction imprecise.

By correcting the B/M values by the estimated average dose these individual differences are partly taken into consideration. The data are less scattered than without correction, but the correlation within the rectum cases is still weak. When correcting the EAD by the 2 Gy ex vivo radiosensitivity factor, a weak correlation with the in vivo irradiation was found for the rectal cancer cohort. But as can be seen this did not eliminate all interfering factors as well and we conclude that there are other interfering factors.

One of these additional factors may be that, even though the cells receive the same dose in each therapy session, the chromosomal aberrations may not increase linear. The breaks per metaphase consist of various aberrations, mostly translocations and dicentrics. The later are an early sensor for acute radiation exposure and increase fast over the first few dose fractions [27–30]. The more dicentric chromosomes accumulate, the more cells go into cell death, as they represent instable aberrations [31, 32]. Most likely only the lymphocytes with stable chromosomal aberrations like translocations are then examined after the 3-week period of radiotherapy. This means not all aberrations induced during therapy are detected. In addition, the patients are irradiated in fractions. The breaks in between the single doses and before taking the blood samples are used by the cells to process induced damages.

Up until now we have not been able to find an algorithm to what extend the number of chromosomal aberrations during treatment can make a statement regarding the individual radiosensitivity. Many studies have, like us, discovered the high interindividual differences that make predictions very difficult [1, 33]. In the previous paragraphs we have indicated, that it is very difficult or nearly impossible to determine the precise dose the blood lymphocytes receive during in vivo irradiation. Additionally it is even more complicated because in vivo there are blood lymphocytes exposed to dosages varying from zero dose up to full dose without knowing which lymphocyte was exposed to which dose. To predict radiosensitivity properly it is extremely important to know the precise dose because only a 10% increase in radiosensitivity will distinctly increase the risk of therapy related side effects. The dose estimation after in in vivo irradiation is certainly less precise than 10%. In ex vivo irradiation using a linear accelerator the dose accuracy is much more precise by about 1–2%.

A clear advantage of using the in vivo irradiation are the physiological conditions in natural environment. Possibly, there is an optimized repair and the resulting data are “closer to the truth”. However even if the ex vivo conditions are less ideal, yet they are highly reproducible. The breaks per metaphase are not an absolute value but a relative measure comparing the B/M of an individual to the Gaussian distribution or rather to the average B/M and a defined threshold. The main advantage of the ex vivo approach are the highly reproducible conditions and this is the advantage that makes it superior to the in vivo data. Our in vitro B/M are only weakly correlated to the ex vivo data. Due to the several disruptive elements in the in vivo irradiation and the highly reproducible condition of the ex vivo approach we conclude that it is extremely difficult to predict radiosensitivity by in vivo IR during therapy.

## Conclusions

Individual radiosensitivity studied by in vivo irradiation is influenced greatly by not exactly definable factors like deposited energy, blood flow, low dose effects and many others. A correlation between individual radiosensitivity estimation studied by ex vivo and in vivo irradiation is definitely unsatisfactory. An estimation of individual radiosensitivity by in vivo irradiation seems not feasible. An ex vivo estimation of individual radiosensitivity should be preferred.

## Data Availability

The datasets during and/or analyzed during the current study available from the corresponding author on reasonable request.
